# Democratising access to dementia research

**DOI:** 10.47795/SQWE8437

**Published:** 2023-11-06

**Authors:** Ruth Dobson, Nirandeep Rehill, Rimona S Weil

## Abstract

As the UK population ages, dementia affects an increasing proportion of the population. There is a drive to accelerate dementia research, however access to research is not equitably distributed. We examine access to dementia research and discuss some enabling factors and barriers. High recruitment is frequently driven by a person (or people) dedicated to improving research participation. Barriers are commonly structural, rather than lack of willingness or knowledge. A recurring issue was lack of time and/or resources. Leveraging existing infrastructure, such as streamlined and efficient governance frameworks, is a clear part of the solution. Research teams need to ensure inclusion/exclusion criteria serve the target population, and that any intervention is accessible to a range of patients. An injection of resources is crucial to support the recruitment process on the ground.

## Background

### The current environment

As the UK population ages, cognitive impairment and dementia affect an increasing proportion of the population. Over 900,000 people live with dementia in the UK; this is expected to increase to >1 million by 2030 [1]. The economic cost of dementia is estimated to triple by 2040 [2]. In response, there has been a drive to accelerate dementia research [3], however, in common with NHS service provision, access is not equitably distributed [4].

Patients living in areas with high socioeconomic deprivation and those from minoritised ethnic groups are less likely to be able to access dementia research. This means that current dementia research, which directs clinical practice, is only really applicable to people of White ethnicity and higher socioeconomic status. However, people living in areas of socioeconomic deprivation have a markedly higher risk of dying with dementia [5] and ethnicity substantially contributes to dementia risk [6] alongside other factors in the recent Lancet commission report [7]. A frequent lack of focus on inclusion of underserved groups has led to a scotoma in knowledge around dementia [8]; the impact of potential interventions on people from underserved groups remains unknown. Understanding the causes, presentations, drivers and modifiers of dementia, along with understanding the impact of interventions in all people with dementia is crucial.

Alongside this, there are key benefits to being treated in a service with an embedded research culture and as well as actively taking part in research. Access to clinical trials of potential disease modifying therapies is the tip of the iceberg. The opportunity to participate in research is an important aspect of care for people living with dementia and provides an opportunity to take control of their condition and a sense of empowerment.

University College London Partners (UCLP) is a partnership that aims to accelerate research and inject innovation into NHS clinical practice. It brings together eight universities, including UCL and Queen Mary University London (QMUL), with NHS Trusts across the South East to a population of 6 million across 26 boroughs. It encompasses a large geographic area from inner city London to coastal Essex, covering five community mental health Trusts (CMHTs) providing memory and dementia services. Mental health Trusts within UCLP deliver services across a range of clinical commissioning groups, serving highly contrasting populations. These include some of the most deprived regions of the country, such as Tower Hamlets, City and Hackney, and Newham, ranked 13th, 15th and 24th in the UK for deprivation. While London has the youngest population nationally, Trusts towards the east of UCLP serve some of the oldest populations around London: Southend-on-Sea, Castlepoint and Rochford, all have a median age above the national average of 40.3 years at 41.8 years, 46.9 years and 46.2 years respectively [9]. Some Trusts serve populations with particularly high proportions from Black, Asian and Mixed ethnicities (see Office for National Statistics Census Maps). It is important to note that NIHR systems used to record and report research participants do not routinely capture ethnicity or deprivation. There is a move to mandate reporting of these data in the foreseeable future. However data flows, security and reporting mechanisms for this sensitive data at provider level are still being established.

## Access to dementia research across UCLP

We sought to understand recruitment to dementia research studies and trials across Trusts within UCLP. Recruitment data demonstrated wide variation in recruitment to NIHR portfolio registered clinical studies (Table 1). These differences become starker when considered in the context of population size, with some of the most successful recruiting Trusts serving the smallest populations. This variation has persisted for a number of years. Although recruitment across all Trusts decreased during the COVID-19 pandemic, high performing Trusts are already showing signs of recovery.

These data include only NIHR portfolio studies; however, these make up the majority of studies within most Trusts. They are not limited to clinical trials (CTIMPS). Fully interventional studies make up around 25% of the total recruited in each Trust; with no substantial variation in proportion of interventional vs non-interventional recruitment between Trusts.

## Understanding the underlying drivers of variation

In order to address this variation and achieve equitable research access, potential reasons for differential participation across Trusts must be understood. In order to scope potential solutions, we undertook focused discussions with clinicians, research managers and service leaders at different levels of seniority at a range of services within UCLP Trusts. We focused on professional groups in order to understand variation in research delivery, as this is an early determinant of unequal access to, and hence participation in, research. As this was a scoping exercise focused on understanding potential barriers and solutions, these discussions were not formally analysed, rather they were used to inform the opinions laid out in this paper.

It is vital to note that addressing barriers to research recruitment requires partnership with underserved communities throughout the research lifecycle, from inception to delivery and interpretation, in order to design relevant interventions that overcome community-specific barriers. There is no “one size fits all”, and interventions need to be targeted at particular services, or even groups within services in order to be successful.

### Enabling factors

In Trusts with high levels of recruitment, there was frequently a person (or people) dedicated to improving research participation. This was either someone directly involved in research programmes, or a person employed for the specific purpose of improving research recruitment. This person was present in clinical environments within the Trust, and usually attended regular clinical meetings.

Successful sites were able to leverage “easy to recruit” studies in order to boost recruitment numbers, and potentially drive resources to sites (“success builds success”). Such studies are often observational, with minimal burden on clinical teams, and generally involve minimal follow-up or intervention. Where visits were required as part of the research process, they would be at times/places convenient to service users. Key infrastructure included “on the ground” factors such as research studies being discussed as a routine and regular part of clinical practice.

Deeper enabling infrastructure elements were a streamlined governance structure across multiple sites. Noclor, a research support service for mental health, community health and primary care, provides a streamlined research governance and facilitation structure across multiple CMHTs. Within East London NHS Foundation Trust (ELFT) a single governance structure allows studies to receive approval across all services within the Trust without the need for site-level agreements. A potential downside is that studies could be adopted without PI engagement, however the clear benefits afforded by the more efficient governance were widely appreciated. We also noted the potential for a virtuous cycle of grant funding and associated metrics flowing to sites as they become more successful.

### Barriers

Barriers were in the main structural, rather than lack of willingness or knowledge. A recurring issue was lack of time and/or resources. In several Trusts, clinical workload meant insufficient time to allow recruitment to studies to be prioritised in any meaningful way. This was coupled with a lack of infrastructure to support recruitment, such as dedicated research staff and/or clinical academics.

At some sites, resource limitation within clinical services, such as inadequate access to imaging for NICE-required standards of dementia diagnosis, meant only a limited number of patients could meet inclusion criteria. Furthermore, the risk of dementia increases with age, and where studies have an upper age limit within inclusion criteria, this can exclude substantial proportions of patients, and subsequently limits applicability of results. Frailty, which is strongly associated with dementia, can limit ability to take part in research studies requiring in person visits. It was also reported that recruitment materials for some studies were inappropriate. Lack of availability of information sheets in languages other than English meant that large numbers of patients were excluded based on language and literacy.

Some more general barriers were an artificial division between mental health and dementia research and a relative lack of institutional experience in the research process leading to time-consuming processes in getting research studies set up. Notably, across different Trusts and levels of service delivery, lack of training or understanding of research was not perceived to be a barrier to research engagement and recruitment.

## How can research involvement be improved across Trusts?

Any intervention needs to address the issues highlighted above, rather than further stretching or dividing services. Service benefits from research take time to accrue, thus enabling interventions need to have rapid impact to produce tangible benefit for people living with dementia and their clinicians. Leveraging existing infrastructure is a clear part of the solution alongside learning from high-performing sites, so that excellence can be emulated.

Better engagement of under-represented groups can also be achieved through infrastructures such as the NIHR Clinical Research Networks (changed to NIHR Research Delivery Networks from April 2024) which coordinate high-quality research through support to research sites. A particular aim of the new NIHR Regional Research Delivery Network is to bring research to under-served regions and communities.

Importantly, more general uptake in dementia research participation will lead to increased recruitment amongst under-served groups. However, to specifically improve equity of access for underserved groups, there is a need for targeted strategies, as highlighted in the recent NIHR-INCLUDE roadmap [8]. These need to be considered by funders and reviewers, in addition to researchers and delivery teams within Trusts. Research teams need to ensure their inclusion/exclusion criteria serve the target population; and that any intervention is accessible to the range of patients in their population. Funders and reviewers should ensure that study outcomes are of relevance to the population being studied; and most important for improving democratisation to research: delivery teams need to identify the under-served groups within their delivery areas (or Trust) as well as the specific barriers to including these groups. It is vital to specifically involve patients from under-served groups in planning potential solutions to widen their participation.

## Conclusion

In order to trigger a step-change in study participation, and thus patient recruitment, an injection of resources is required, most likely in the form of delivery teams. These teams would ideally be supported via the CRN or other external infrastructure, to support the recruitment process on the ground. Given positive clinical trials of disease modifying therapies in dementia, there is the potential that services will undergo substantial and rapid change, which may bring opportunity for research delivery. However, the number of people eligible for any disease modifying therapy is likely to be a fraction of those living with dementia, which has the potential to drive further inequity. Should such therapies reach clinical practice, patient selection and treatment pathway will be via regional centres, probably led by or run jointly with neurology. There is a real risk that this further entrenches the division between relatively well-funded neurological research centres that operate in close proximity to major universities and community mental health units that do the majority of dementia diagnosis but are situated in community settings.

Equitable patient involvement in research does not just mean taking part in research. Involvement of representative populations throughout the research process is required to build trust; working in partnership with a range of patient groups to ensure that research questions and approaches have relevance to all is crucial. Work to improve the cultural competence of researchers designing and setting up studies will ensure that approaches are grounded in meaningful dialogue with individuals and communities. Particular areas of need are around active recruitment to studies together with clinical follow-up, given currently overstretched clinical teams. Ideally, research teams would undergo training at well-performing sites, sharing established knowledge and patterns of effective recruitment strategies. Alongside this, support for new academic posts, research-active clinicians such as psychiatrists and associated healthcare professionals able to drive research and act as study principal investigators, as well as the environments and infrastructure to nurture them will provide an on-going source of potential research instigators.

Approaches such as this will lead to not only higher recruitment across geographical locations, and diverse populations, but will also translate into improved clinical outcomes, especially in under-served groups through more closely embedding research into clinical practice. It will allow clinicians and researchers working to improve access to research for all patients, and facilitate the study of dementia in people from all backgrounds.

## Figures and Tables

**Table 1:** Recruitment to NIHR portfolio studies by CMHT. CMHTs are ordered by size,from largest to smallest population size. The COVID-19 pandemic placed restrictions on study recruitment from the start of financial year 2020-21. NIHR, National Institute for Health and Care Research, CMHT, community mental health trust; NELFT, North East London NHS Foundation Trust; EPUT, Essex Partnership University NHS Foundation Trust; ELFT, East London NHS Foundation Trust; BEH, Barnet, Enfield and Haringey Mental Health Trust; C&I, Camden and Islington NHS Foundation Trust.

Financial year																
Name of Trust, population	2014-15		2015-16		2016-17		2017-18		2018-19		2019-20		2020-21		2021-22*	
	S	R	S	R	S	R	S	R	S	R	S	R	S	R	S	R
NELFT (c 4.3 million)	3	19	9	246	12	799	13	396	82	279	10	82	51	98	51	62
EPUT (c 2.5 million)	3	19	-	-	-	-	9	150	6	48	7	110	3	10	5	191
ELFT (c 1.4 million)	3	121	4	25	-	-	-	-	1	<5	-	-	2	11	1	15
BEH (c 1.2 million)	4	52	4	20	2	28	4	36	7	44	5	36	4	24	3	28
C&I (C 0.5 million)	6	1694	6	387	7	955	9	672	14	248	9	192	5	54	4	96

* to end March 2022

S=number of studies; R=absolute number of patients recruited.

## Data Availability

Recruitment numbers presented in this manuscript were obtained from open databases available via www.nihr.ac.uk.
